# Vibrotactile Alerting to Prevent Accidents in Highway Construction Work Zones: An Exploratory Study

**DOI:** 10.3390/s23125651

**Published:** 2023-06-16

**Authors:** Xiang Yang, Nazila Roofigari-Esfahan

**Affiliations:** 1 Department of Industrial and Systems Engineering, Virginia Tech, Blacksburg, VA 24061, USA; xiangyang@vt.edu; 2 Department of Building Construction, Myers-Lawson School of Construction, Virginia Tech, Blacksburg, VA 24061, USA

**Keywords:** highway work zone, construction worker, safety, vibrotactile warning

## Abstract

Struck-by accidents are the leading cause of injuries in highway construction work zones. Despite numerous safety interventions, injury rates remain high. As workers’ exposure to traffic is sometimes unavoidable, providing warnings can be an effective way to prevent imminent threats. Such warnings should consider work zone conditions that can hinder the timely perception of alerts, e.g., poor visibility and high noise level. This study proposes a vibrotactile system integrated into workers’ conventional personal protective equipment (PPE), i.e., safety vests. Three experiments were conducted to assess the feasibility of using vibrotactile signals to warn workers in highway environments, the perception and performance of vibrotactile signals at different body locations, and the usability of various warning strategies. The results revealed vibrotactile signals had a 43.6% faster reaction time than audio signals, and the perceived intensity and urgency levels on the sternum, shoulders, and upper back were significantly higher than the waist. Among different notification strategies used, providing a moving direction imposed significantly lower mental workloads and higher usability scores than providing a hazard direction. Further research should be conducted to reveal factors that affect alerting strategy preference towards a customizable system to elicit higher usability among users.

## 1. Introduction

### 1.1. Background

Fatal and nonfatal occupational injuries remain prevalent, with 2.6 million nonfatal and 5190 fatal occupational injury cases being reported in the United States in 2021 [1]. Among all job sites, construction environments remain one of the most dangerous working environments [2]. Between 1992 to 2015, there were a total of 25,705 fatalities resulting from work-related injuries in the construction industry, averaging at about 1,071 deaths annually [3]. Traditional interventions such as organizational interventions, safety programs, incentives, and legislation have been proposed to address safety concerns, but their effectiveness in the construction industry remains uncertain [4,5]. In 2021, there were 72,800 nonfatal and 986 fatal injuries in the construction industry, resulting in a total cost of USD 167 billion [6]. Among all construction sectors, highway work zones are one of the most dangerous work sites, with 29,493 individuals losing their lives in work zone crashes from 1982 through to 2020 (about 776 per year) [7]. Struck-by injuries with passing vehicles or heavy equipment were the leading causes of nonfatal injuries and the second most common cause of fatalities [8]. As such, it is essential to improve the safety of construction workers in highway work zones who are exposed to struck-by incidents.

Numerous technologies have been developed to enhance safety at highway construction work zones [9], emphasizing speed reduction [10,11], intrusion prevention [12], intrusion alert [13], and proximity hazard warning systems [14]. Exposure to live traffic is often unavoidable in highway work zones, and providing alerts can be a cost-effective way to reduce struck-by injuries. Intrusion and proximity hazard detection technologies use active strategies to provide visual and auditory warnings to enhance workers’ awareness of hazards [14,15]. These warnings can be conveniently integrated into existing user interfaces such as visual displays and speakers. For instance, Burkett and Velinsky [16] evaluated an intrusion alert system that uses an audible alarm (typically 120–130 dB) to notify workers in the immediate vicinity when a vehicle intrudes the work zone. Banaeiyan et al. [17] developed a visual-based warning system that provides visual alerts to construction vehicle operators using a tablet installed in the vehicle.

Although these systems demonstrate robust functionality, the efficacy of visual or audio alerts in highway construction work zones can be reduced by visibility issues and high noise levels in those environments. These work zone environmental conditions can significantly hinder a worker’s perceptions of visual and auditory warnings in urgent situations [18,19]. Therefore, visual and audio signals may not be optimal to provide warnings and navigation cues to workers in complex highway work zone environments.

Furthermore, based on Wickens’ Multiple Resource Theory (MRT) [20,21,22,23,24], cross-modal time sharing is more effective than intramodal time sharing. In other words, receiving information in different sensory modalities can result in faster responses and higher accuracy levels compared to receiving information in a single sensory modality. Previous studies have also indicated that cross-modal displays outperform intramodal displays [25]. This suggests that providing alerts through a sensory modality other than vision or audio could be more effective, especially in life-threatening situations where response time and correctness are crucial.

### 1.2. Haptics and Vibrotactile Systems

Haptics, or the sense of touch, has been studied as a communication channel since 1957 [26]. It involves perceiving kinesthetic (force/position) and cutaneous (pressure, vibration, temperature, and pain) sensations through skin receptors [27,28,29]. Perceiving haptic information is independent of visual or auditory inputs, making it a promising sensory channel to receive alerts in noisy and visually cluttered environments.

Displays that convey information through haptics are often referred to as tactile displays. They can be categorized into vibrotactile, electrotactile, and thermal displays, depending on the actuation source [28,30,31]. Vibrotactile displays are particularly advantageous in terms of safety, cost, and ease of implementation. They use vibration motors to stimulate cutaneous receptors in the skin to transmit the planned information. By adjusting the frequency, amplitude, pulse rate, and contact location of vibration motors on the skin, different types of information can be conveyed. Therefore, vibrotactile displays offer the potential to display various types of information.

Vibrotactile displays are widely used in military, rehabilitation, driving, sports, and occupational settings [32,33,34,35,36,37,38]. They are primarily used for navigation, motor learning, and event triggering [31,39]. The independence of a vibrotactile display enables alerts, spatial orientation, and guidance to be perceived even in complex environments with loud noise and limited visibility [40]. For instance, Erp et al. [41] and Faugloire et al. [42] studied the effectiveness of using vibrotactile waist belts as navigation tools, with eight vibration motors positioned equidistantly around the waist to indicate the target direction. Although these two studies mapped vibration and directional cues differently, both concluded that vibrotactile waist belts can effectively provide directional information with high accuracy and low deviations.

Vibrotactile systems have also been studied for their potential to improve safety in construction highway work zones [32]. Cho and Park [43] developed and tested a vibrotactile system that alerts construction workers about upcoming hazards. Four vibration motors were placed on the lower back of users to alert them about upcoming hazards. Participants were given three moving commands, “Move Right”, “Move Left”, and “Sit Down”, which were mapped with three vibration intensities and three vibration durations. The study found that the overall failure rate was less than 6%, indicating that vibration intensities and signal lengths can deliver hazard information effectively in urgent situations. In a later study, Sakhakarmi et al. [44] evaluated the effectiveness of a vibrotactile system that helps construction workers avoid being struck by vehicles in roadside work zones. They placed ten motors on the lower backs of participants and mapped eight hazard directions, three hazard levels, and two types of vehicles. The results from a field test with five participants showed that 99.12% of hazard directions could be correctly identified when received as the only information provided to them. However, the correctness of identifying hazard direction decreased to 97.52% when the hazard level and equipment type were also provided. This suggests that it is essential to avoid providing too much information to the users.

Vibrotactile systems enable construction workers to receive hazard information even in noisy, vision-obstructed environments. However, current approaches only consider one body part for receiving vibrotactile alerts and fall short in considering realistic situations where workers should respond to warnings as a secondary task while engaged in their primary work task. Additionally, human factors involved in the perception of various information through vibrotactile signals are overlooked. This study aims to add to the current body of knowledge and make full benefit of the advantages of vibrotactile systems in improving safety in highway work zones by addressing the following research questions: 1. What are the optimum locations to place vibration motors on construction workers’ bodies to maximize the efficacy of the provided warnings, considering their necessary apparel [32]? 2. Are vibrotactile systems effective when workers are actively engaged in their work tasks? 3. How should human perception and preference be considered in the design of vibrotactile systems aiming to provide alerts in dangerous situations?

To answer these questions, this study took an innovative approach by considering various human factors in both the system design and experimental analysis. As a primary measure, the positioning of the vibration units was inventively configured and evaluated to augment the intuitiveness of the vibration signal for users. The system also incorporated a state-of-the-art vibration motor that yielded considerably higher vibration intensity without causing annoyance. Such a feature guarantees the perceivability of haptic warnings in urgent situations, even under circumstances where users’ attire might attenuate the vibrations. Furthermore, the study went beyond current approaches by evaluating and comparing user experiences with different notification strategies, leading to identifying the most effective strategy for different situations. Additionally, to assess the usability and effectiveness of vibrotactile systems and evaluate how the surrounding environment can hinder construction workers’ perception of warnings, the participants of the research were exposed to realistic yet safe highway working environments through 360° videos presented in Virtual Reality (VR). Lastly, in the experiment design, participants were asked to conduct construction work in VR as the primary task, while responding to vibrotactile alerts served as the secondary task. This involved the human factor consideration of the task-switching process [24], which engendered a more authentic working routine and enabled the realistic evaluation of the system’s usability. In conclusion, these methodological approaches facilitated a more practical and integrative assessment of the system’s performance and user engagement.

### 1.3. Research Objective

The goal of this study was to investigate the usability of a vibrotactile warning system integrated into construction workers’ personal protective equipment (PPE), i.e., safety vests. The proposed system features a new motor layout and motor type, taking into account human factors such as ease of use, users’ perception of vibrations, workload, and the intuitiveness of the mapping strategy between the provided information and vibration patterns. The study aimed to ensure that the vibrotactile system could effectively provide alerts and navigation guidance to users in hazardous situations. To achieve this goal, the following objectives were addressed:

1. Assessing the feasibility of using vibrotactile signals compared to visual and audio signals to provide warnings and hazard avoidance guidance in highway construction work zone environments.
2. Investigating the perception and performance of vibrotactile signals activated at various body locations.
3. Evaluating the usability of different notification strategies, e.g., presenting a moving direction (navigation) vs. hazard direction, in warning workers of imminent threats.

## 2. Materials and Methods

This study was conducted in three phases: Phase 1 aimed to compare the reaction times in response to visual, audio, and vibrotactile warning signals within a virtual construction environment to assess the viability of using vibrotactile signals as warnings in highway construction work zones. In phase 2, a vibrotactile system was proposed to deliver vibrations at six body locations: sternum, left/right waists, left/right shoulders, and upper back. This phase focused on evaluating the perception and reaction time of users to vibrotactile signals located at various body parts, providing insights into the design of viable wearables that can be used conveniently and efficiently by highway workers. In phase 3, three notification strategies were explored to avoid hazards: providing a moving direction, providing a hazard direction, and providing a hazard direction followed by a moving direction. This phase sought to compare the efficiency of distinct notification strategies to convey directional cues to users through haptics and present a comprehensive evaluation of the proposed system and various notification strategies. In each phase, user studies were conducted to collect feedback regarding these different features, as explained below.

### 2.1. Participants

A sample of 17 healthy participants (12 males and 5 females) aged between 18 and 45 was recruited from college students. The participants had a mean ± standard deviation age of 26 ± 3 years and body mass of 76 ± 18 kg. Participants were required to have the ability to walk without assistance and feel comfortable using a VR device. All participants had no previous experience of using vibrotactile systems. Prior to the start of the experiments, informed consent forms and demographic questionnaires were provided to participants in both electronic and paper-based formats. The study was approved by the institutional review board of Virginia Tech.

### 2.2. VR Headset and Virtual Environment

**Figure 1:**
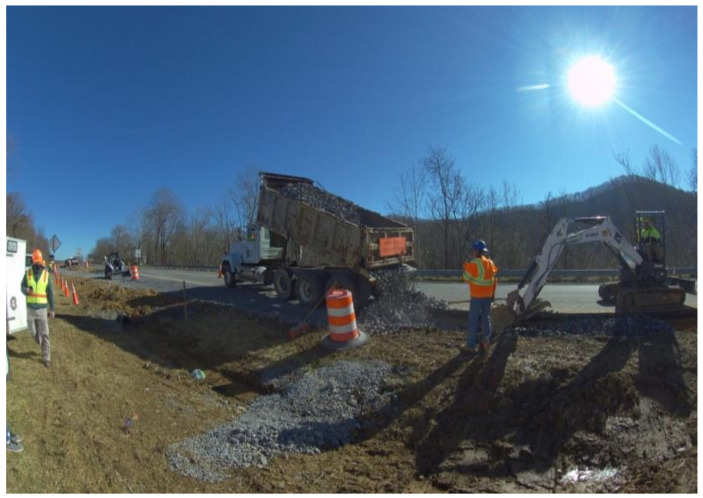
A 360° video image of highway construction work zone.

This study utilized Virtual Reality (VR) to simulate an actual highway environment, employing 360° videos of actual highway work zones presented via head-mounted displays (HMDs), to provide users with a thoroughly immersive experience and enhance their sense of presence in the environment [45]. Participants used a Pico Neo 3 Pro VR headset to play 360° video footage recorded from multiple highway construction work zones (Figure 1). Contrasting Kim et al.’s [46] and Jelonek et al.’s [47] game-like virtual environment, the pre-recorded 360-degree video footage employed in this study created a more realistic environment for participants while they performed various tasks during the experiment. Being a stand-alone device, the Pico VR headset allowed the users to move freely within a circled area of a three feet radius. Additionally, the headset had two 3D spatial speakers located near the ears, which enabled participants to be immersed in realistic spatial audio usually present at highway work zones, e.g., the noise of construction machinery and passing traffic, while still being aware of their surroundings.

### 2.3. Phase 1: Reaction Time of Different Sensory Modalities

The first phase of the study aimed to compare users’ reaction times in response to visual, audio, and vibrotactile warning signals in a virtual construction environment. Reaction time was measured as the shortest duration from receiving the warning signal to the start of the user’s action to move towards a safe area. The experiment was designed to determine if there was a significant difference in reaction time for visual, audio, and vibrotactile warning signals, considering the conditions of highway environments such as high noise and ambient light.

#### 2.3.1. System Components

**Figure 2:**
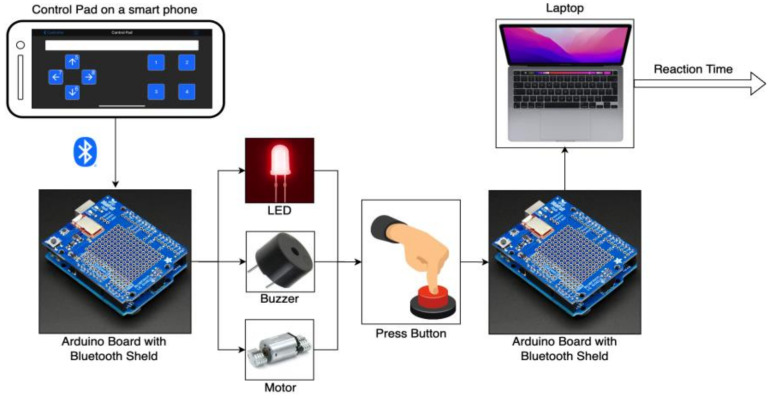
The system setup for calculating the reaction time of different sensory modalities.

**Figure 3:**
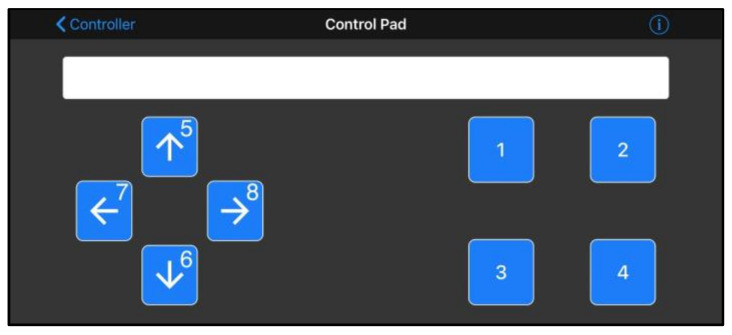
Control Pad in the BluefruitConnect application. The four buttons on the left side, marked with arrows, were programmed to activate vibration motors located on SV to provide the corresponding directions. The four buttons on the right side were utilized to select the desired vibration setting for the motors.

Figure 2 presents an overview of the proposed system designed to test the reaction time of different sensory modalities. The system delivers warning signals through three sensory modalities: visual, audio, and vibrotactile. It comprises a red LED light (1.8 V), a passive buzzer (85 dB, 2.3 kHz @ 5 V), a vibration motor (8000 rpm, 15 G @ 3.7 V), an Arduino UNO R3, a smartphone, and a Bluetooth board. Wireless communication between the Arduino board and the smartphone is facilitated by the Bluetooth board. To initiate the system, a Bluetooth connection is established between a smartphone and the Arduino board through an application called “BluefruitConnect”. The application features a Control Pad that enables the user to send a signal to the Arduino board wirelessly by tapping one of the 8 buttons (as illustrated in Figure 3). The functionality of buttons was modified in three different phases. In this phase, tapping “1”, “2”, or “3” will activate the red LED light (visual warning), the passive buzzer (audio warning), or the vibration motor (vibrotactile warning), respectively.

**Figure 4:**
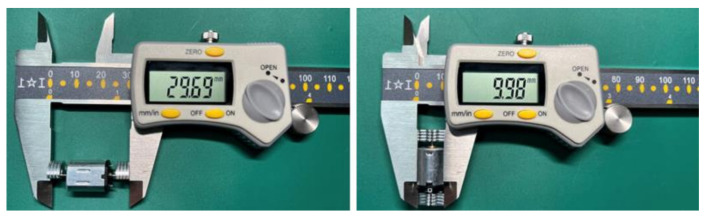
Double-headed vibration motor [54].

The vibration motors (see Figure 4) utilized in this study were eccentric rotating mass (ERM) motors. These motors are designed to rotate continuously when a voltage or current is applied, with each motor containing a 3.7 V DC motor and an off-center mass attached to each side of the output shaft. The motors rotate at 8000 rpm, generating a vibration amplitude of 15 G under the rated voltage, where 1 G is equivalent to the acceleration from gravity. In previous studies, smaller vibration motors (approximately 10 mm in size) have often been utilized as they have small dimensions and weights that allow for the formation of a motor array attached to the human body [48,49]. For example, coin-shaped motors with a diameter of 12 mm and thickness of 3 mm can generate 2 G acceleration at 3.7 V [50], while cylindrical motors with a diameter of 7 mm and a length of 16 mm can create a maximum acceleration of 7.5 G [51]. Based on the literature and our preliminary tests, these motors work effectively when directly attached to the skin or over thin cloths [52,53].

This system records and calculates the time difference between two events: the activation of a warning signal and the user pressing a button. In this experiment, visual warning signals were provided by the red LED light mounted on the VR headset between the eye lenses, which was visible when the VR headset blocked users’ vision of the real environment. Audio warning signals were provided by the buzzer located at the waist of the user, while vibrotactile warning signals were provided by the vibration motor located at the bottom of the sternum of each participant.

#### 2.3.2. Experimental Design

In this experiment, the order of three types of warning signals was randomly assigned and counterbalanced in 15 trials for each participant. This ensured that each type of warning signal was triggered 5 times in a random order, minimizing the possibility of order effects or bias.

#### 2.3.3. Experimental Procedures

At the beginning of the experiment, participants were asked to stand still and put on the VR headset. The 360° video would then begin playing, and participants were instructed to observe a construction worker’s movements as if they were new workers on the construction site, shadowing experienced workers. Each trial began with the researcher standing about 1 m away and pressing a number key (1–3) on the Control Pad (Figure 3) to activate one of the warning signals. After a random delay period (1~1.5 s) generated by the system, the warning signal was activated. Participants were instructed to press a button as soon as they noticed the warning signal. The system then recorded the time duration between the activation of the warning signal and the button press.

### 2.4. Phase 2: Vibrotactile Signals at Different Body Locations

To evaluate the efficacy of vibrotactile signals at various body locations, this experiment incorporated two tasks: a perception task (task 1) and performance task (task 2). Task 1 sought to gather participants’ subjective perceptions of distinct vibration types at each body location, offering a foundational understanding of their perception of vibrotactile signals and facilitating the interpretation of subsequent tasks. Task 2 aimed to compare participants’ reaction times to vibrotactile signals presented at different body locations and examine whether the effectiveness of alerting users was consistent across various body locations for vibrotactile signals.

#### 2.4.1. System Components

**Figure 5:**
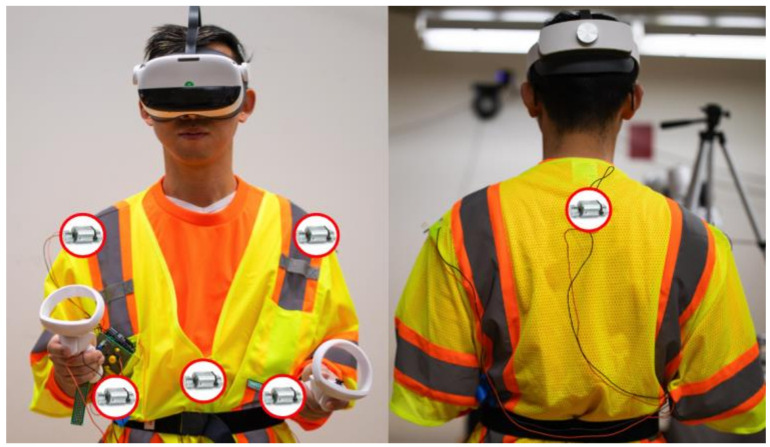
Placement of the vibration motors on the Smart Vest.

In phase 2, a second system was designed to be attached to workers’ conventional PPE, called a “Smart Vest” (SV). The layout enabled the delivery of vibrotactile warning signals via six vibration motors (8000 rpm, 15 G @ 3.7 V) situated at six distinct body locations (Figure 5): sternum, left/right waists, left/right shoulders, and upper back.

**Figure 6:**
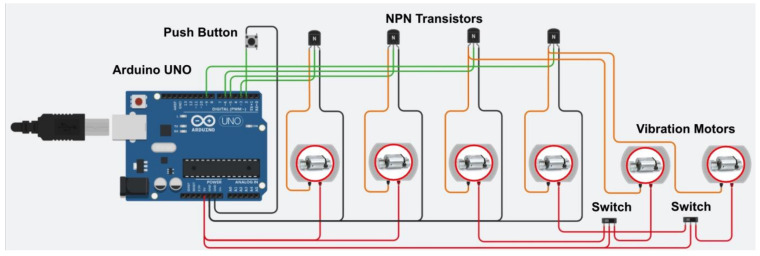
Electrical design of the Smart Vest.

The primary hardware components of the SV system consist of six vibration motors (Figure 4), an Arduino UNO R3, a smartphone, and a Bluetooth board. Figure 6 illustrates the instrument model the SV is illustrated in. The control of six vibration motors was achieved through four output pins on the Arduino board, with each pin being associated with one of four designated directions. To evaluate the effectiveness of vibrotactile signal delivery at both the shoulder and waist locations, vibration motors were placed bilaterally on these two sites. For a convenient transition between these two regions, the system is equipped with two switches, each being dedicated to the left and right side. The rotation speed of the motors was regulated through four NPN transistors using the pulse-width modulation (PWM) method, as cited in reference [55]. To provide manual control over the cessation of vibrations, a push button was integrated into the system. In Figure 6, green lines represent the wires used for sending controlling signals; black lines represent wires connected to ground pins on the Arduino board; orange lines represent wires connected between NPN transistors and motors; and red lines represent wires connected to the power supply pin (5 V) of the Arduino board. The Bluetooth board was installed on top of the Arduino board, which enables wireless communication between the Arduino board and a smartphone. To initiate the system, the SV system first connects with a smartphone through the BluefruitConnect application. Researchers then utilize the Control Pad (Figure 3) to send commands to the Arduino board via Bluetooth. By uploading various programs to the Arduino board, researchers can manipulate the intensity, pulse length, and locations of vibrations on the SV.

To improve user convenience, vibration motors were attached to the safety vest directly. In the construction industry, workers are required to wear safety vests over their daily clothing [56], so integrating the vibrotactile system into the safety vest eliminates the need for workers to put on additional apparel, which can impose additional burden. There are multiple challenges in selecting suitable vibration motors for a wearable device such as the SV, designed for use in construction environments. First, with the presence of extra clothing layers, ensuring that vibrations are perceivable by users can be challenging. Second, the selected motors should be light, so they do not add burden to the wearer and do not cause discomfort when conducting various activities. Various sizes of vibration motors, producing different vibration intensities, were tested, and it was determined that the double-headed vibration motor provides an acceptable balance between size and vibration amplitude. To ensure close contact between the motors and the user’s trunk, an elastic waist belt was attached around the user’s waist and adjusted to their preferred level of tightness for comfort.

Various human factors were considered when selecting the candidate locations for the vibration motors on the SV. A common method for conveying directional information involves using a belt to securely attach vibrotactile actuators around the waist at equal intervals [57,58,59]. Some studies have also positioned motor arrays on the lower back to provide additional information beyond directional cues [44,49]. In these studies, participants were able to distinguish vibrations from different motors placed 40 mm apart. Although Jóhannesson [60] reported that the torso’s spatial acuity was below 13 mm, research by Cholewiak et al. [61] suggests that reducing the number of motors and increasing the distance between vibrotactile sites can significantly enhance the ability to localize vibration bursts. Consequently, to minimize the number of motors on the SV to reduce the added weight, four pieces of directional information (i.e., front, back, left, and right) were considered to be conveyed through haptic signals. The preliminary tests of this study indicated that vibrations delivered to the left and right sides of the waist and the lower back were occasionally challenging to perceive, even with the waist belt. Several other studies have demonstrated that vibrations provided on the sternum, shoulders, and upper back [29,33,39,62] can be reliably perceived. Consequently, six locations (i.e., sternum, left/right waist, left/right shoulder, and upper back) were considered as candidate locations to receive vibrotactile signals on the SV system.

#### 2.4.2. Vibration Pattern Design

In this experiment, motor locations, vibration intensities, and pulse lengths were incorporated as independent variables. Two vibration intensities (50% and 100% of the maximum intensity) were included to evaluate the perceivability of the vibrations. Two pulse lengths (60 ms and 400 ms) were chosen based on several previous studies and the preliminary tests in the current study. It was discovered that the vibration pulse lengths below 60 ms were sometimes not perceivable. Cho and Park [43] also noted that pulse lengths below 50 ms were not perceivable. One possible reason is that ERM motors need more than 50 ms to reach the perceivable vibration magnitude. Another pulse length, 400 ms, was selected based on an earlier study by Pratt et al. [63]. They found that when the pulse length was near 400 ms, participants’ mean perceived urgency, annoyance level, and acceptability ratings were all at the midpoint of the scale (50 out of 100). Consequently, this pulse length was adopted in this study and considered as a baseline. Furthermore, to make vibration patterns more distinguishable, the pulse length and inter-pulse length were set to be the same in this experiment, as participants are sensitive to both pulse length and inter-pulse length [64].

#### 2.4.3. Perception Task: Experimental Design and Procedures

In the perception task, vibrations with combinations of 4 directions (front, back, left, and right), 2 vibration intensities (50% and 100% of the maximum intensity), and 2 pulse lengths (60 ms and 400 ms) were activated in random order. In total, 16 trials (4 directions × 2 intensities × 2 pulse lengths) were conducted. To compare the perception of vibrations on shoulders and waists, these trials were conducted for both setups. The perceived intensity and urgency level of the vibration were measured as dependent variables. Additionally, participants were asked to report their response to the perceived directional information for each vibration. For example, when the motor located at their sternum (front) was activated, some participants reported that they would go forward, and others reported that they would go backward.

In this task, participants started by watching 360° videos of a highway construction work zone on the VR device. Participants were required to follow one of the construction workers’ movements as if they were working on the construction site. When the participants were ready to receive the vibrotactile signals, the researchers stood approximately one meter away and activated a motor with a certain combination of pulse rate, intensity, and location. In each trial, only one type of vibration was activated. When a participant confirmed that he/she noticed the vibration, he/she was asked to report the location of activated motors and their perception of the vibration in terms of perceived intensity and urgency level.

#### 2.4.4. Performance Task: Experimental Design and Procedures

In the performance task, vibrations were activated in a random order, with combinations of 4 directions (front, back, left, and right), 2 vibration intensities (50% and 100%) at the rated voltage, and 2 pulse lengths (60 ms and 400 ms). In total, 16 trials (4 directions × 2 intensities × 2 pulse lengths) were conducted. To compare the performance of vibrations on the shoulders and waist, these trials were executed for both setups. The reaction time of each vibration was measured as the dependent variable.

In this task, participants began by watching the 360° video of a highway construction work zone on the VR device. They were required to follow one of the construction workers’ movements as if they were working on the construction site. When participants were ready to receive the vibrotactile signals, the researcher stood approximately one meter away and activated the developed program for the performance task. Once the researcher selected a certain combination of pulse length and location for the vibrotactile signal, the assigned motor was activated after a random period between 1 and 1.5 s. Participants were instructed to press a button as soon as they noticed a vibration signal. Reaction time was calculated by subtracting the timestamp of pressing the button from the timestamp of the vibration activation. The reaction time of the vibrotactile signals was recorded after each trial.

### 2.5. Phase 3: Usability of Different Notification Strategies

To determine an effective notification strategy for sending directional cues to users through vibrotactile alerts, two tasks were conducted in this experiment: the drawing task (task 1) and the moving task (task 2). Task 1 was adapted from a study by Li et al. [53], in which participants were asked to draw a continuous pathway on a grid paper based on the vibrotactile signals they received. However, this study went beyond the scope of Li et al.’s research by immersing the participants in highway scenarios and assigning a primary task of following worker activities to replicate realistic highway work zones. Because their vision was obstructed by the VR device, they were asked to draw a straight line in the direction of movement on an iPad for each vibration. Task 2 was modified from a study by Erp et al. [41], in which participants were instructed to move toward the indicated direction based on the vibrotactile signals they received. In this study, to ensure safety in the lab environment, the participants were asked to take one step in response to the vibrotactile signal and then return to the starting location afterward. The aim of these two tasks was to examine the usability of three different notification strategies provided by the SV system.

#### 2.5.1. System Components

In this experiment, the SV system was utilized to deliver vibrotactile signals, and an iPad with an Apple Pencil was used to record drawings. The SV system used in this phase was the same as the one employed in phase 2; however, it was loaded with a different Arduino program designed to provide directional cues using three distinct notification strategies through vibrations.

#### 2.5.2. Notification Strategies

Three notification strategies were examined in this experiment:

1. Providing moving direction: This strategy was designed to guide workers towards a safe location in hazardous situations. As such, the motor located in the moving direction was continuously activated with a 400 ms pulse and a 400 ms inter-pulse period. This strategy indicated the direction participants needed to move towards to avoid the hazard. It was designed based on the concept of vibrotactile navigation systems [41,53].
2. Providing hazard direction: This strategy indicated the direction of the hazard, so participants needed to move in the opposite direction of the activated motor. The motor located in the hazard direction was activated continuously with a 60 ms pulse and a 60 ms inter-pulse period. It was designed based on the concept of vibrotactile warning systems [44,65,66].
3. Providing hazard direction and then moving direction: This strategy combined both strategy 1 and strategy 2, aiming to provide both hazard directions and moving directions. To this end, first, the motor located in the hazard direction was activated with a 60 ms pulse and a 60 ms inter-pulse period three times. Then, the motor located in the moving direction was activated continuously with a 400 ms pulse and 400 ms inter-pulse period.

#### 2.5.3. Experimental Design

In this experiment, for each notification strategy, vibrations were activated in combinations of 4 directions (front, back, left, and right) with 2 repetitions in each direction, in random order. To compare the efficacy of vibrations on shoulders and waists, 8 trials (4 directions × 2 repetitions) were conducted for both setups for the same strategy. In total, 48 trials (3 strategies × 4 directions × 2 repetitions × shoulder/waists) were performed in both the drawing and moving tasks for each participant. The order of three strategies was randomized for all participants to minimize the possibility of order effects or bias.

The dependent variables measured in this experiment included the correctness of following the directional cues, NASA-TLX workload, usability ratings (comfort level, effectiveness, and willingness to use the strategy), and preferred strategy. NASA-TLX is a standardized multidimensional scale designed to evaluate the workload for a task. The measurements include mental demand, physical demand, temporal demand, effort, performance, and frustration level [67]. Each measurement is assigned a weight by asking participants to compare the importance of each pair of them and then assign a rating (0~10) to each.

#### 2.5.4. Experimental Procedures

A brief familiarization period preceded the data collection for each strategy, allowing participants to acclimate to the current strategy.

In the drawing task, participants began by watching the 360° video of a highway construction work zone on the VR device and following one of the construction workers’ movements. During each trial, a researcher stood approximately one meter away and activated one of the six vibration motors on the SV. As soon as participants perceived a vibrotactile signal, they were required to draw a straight line on the iPad, indicating the direction they needed to move towards. Their drawings were screen-recorded for further analysis. Upon completing a drawing, participants pressed a button to stop the vibrotactile signals. After finishing 16 trials for a strategy, participants completed a NASA-TLX form to report their workload while using that strategy.

In the moving task, participants started by watching the 360° video of a highway construction work zone on the VR device and following one of the construction workers’ movements. During each trial, a researcher stood approximately one meter away and activated one of the six vibration motors on the SV. As soon as participants perceived a vibrotactile signal, they were instructed to take one step towards the safe area. Their movements were video recorded for further analysis. Upon reaching the safe area, participants pressed a button to stop the vibrotactile signals and returned to the starting location. After completing eight trials for a strategy, participants filled out a NASA-TLX form to report their workload while using that strategy.

Once all tasks were finished, participants were asked to complete a questionnaire about the usability of three different strategies and to indicate their most preferred strategy.

### 2.6. Data and Statistical Analysis

All statistical analyses were conducted using JMP Pro 16. Statistical significance was examined at the conventional level of *α* = 0.05 for all tests.

#### 2.6.1. Reaction Time for Different Sensory Modalities

The mean ± standard deviation (SD) reaction time for each sensory modality was calculated across all participants. Separated repeated-measures analyses of variance (ANOVAs) [68] were conducted to assess the main effects of sensory modality on reaction time. Participants were included as a blocking effect in these ANOVAs. Significant main effects were further examined using Tukey’s HSD post hoc tests [68].

#### 2.6.2. Reaction Time for Different Body Locations

The mean ± SD reaction time for each motor location was calculated across all participants. Separate repeated-measures ANOVAs were used to compare the effect of the motor location on reaction time. Participants were included as a blocking effect in the ANOVAs. Significant main effects were examined further using Tukey’s HSD post hoc tests. Analyses for vibrations with 60 ms and 400 ms pulse lengths were evaluated separately.

#### 2.6.3. Correctness

The mean ± SD correctness for identifying locations of activated motors and following the directional cues was calculated across all participants. Separate repeated-measure ANOVAs were used to compare the effect of the notification strategy and task type on correctness. Participants were included as a blocking effect in the ANOVAs.

#### 2.6.4. Perception

The mean ± SD reaction time for each motor intensity, pulse/inter-pulse length, and each motor location were calculated across all participants. Nonparametric Kruskal–Wallis tests [68] were conducted to determine the effects of motor intensity, pulse/inter-pulse length, and body location on perceived intensity and urgency level. A simple linear regression was used to test if perceived intensity significantly predicted urgency level.

#### 2.6.5. NASA-TLX Workload

The mean ± SD workload for each notification strategy was calculated for both the drawing task and the moving task across all participants. Separate repeated-measures ANOVA was used to compare the effect of the notification strategy on workload. Participants were included as a blocking effect in the ANOVAs. Significant main effects were examined further using Tukey’s HSD post hoc tests.

#### 2.6.6. Usability

Nonparametric Kruskal–Wallis tests were performed to find the effect of strategy on comfort level, effectiveness, and willingness to use the strategy. To understand the overall usability of each strategy, the usability ratings were summed up as the usability score for each participant. A nonparametric Kruskal–Wallis test was used to find the effect of strategy on the usability score.

## 3. Results

### 3.1. Reaction Time for Different Sensory Modalities

**Figure 7:**
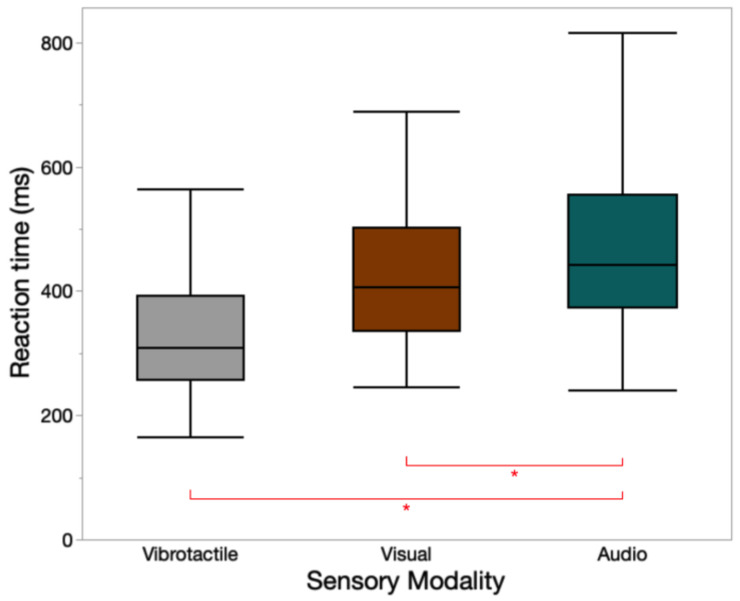
Reaction time of different sensory modalities. * indicates significant pairwise Tukey HSD comparisons (*p* < 0.05) among the sensory modalities.

A total of 255 trials (17 participants × 15 trials/participant) were conducted in this task. The mean ± standard deviation (SD) values of reaction time for vibrotactile, visual, and audio signals were 337 ± 116 ms, 443 ± 153 ms, and 597 ± 639 ms, respectively. Sensory modality had a significant main effect on the reaction time (*F*(2, 222) = 10.24, *p* < 0.001). The Tukey HSD post hoc tests indicated warning signals sent through vibration and light have significantly shorter reaction times than those sent through sound. A box plot of reaction time for three types of warnings is presented in Figure 7.

### 3.2. Reaction Time for Different Sensory Modalities

A total of 544 trials (17 participants × 16 trials/participant × 2 pulse length) were conducted in this task. The mean ± SD of reaction time are listed in Table 1 and Table 2. No significant differences were observed between different motor locations for vibrations with either 60 ms pulse length (*p* = 0.025) or 400 ms pulse length (*p* = 0.065).

### 3.3. Correctness

A total of 1088 trials (17 participants × 64 trials/participant) were conducted in phase 2. Participants were able to identify the locations of activated motor locations with 100% correctness.

A total of 816 trials (17 participants × 48 trials/participant) were conducted in phase 3. The mean ± SD values of correctness in responding to warning messages were 95.77% ± 1.08%. No significant differences were observed between different notification strategies (*p* = 0.899) or types of tasks (*p* = 0.893).

### 3.4. Perception

A total of 544 trials (17 participants × 32 trials/participant) were conducted in this task. The mean ± SD values of perceived intensity (rating from 0~10) and urgency level (rating from 0~10) for different motor intensities (50% and 100%) and pulse lengths (60 ms and 400 ms) are listed in Table 3. The mean ± SD values of perceived intensity and urgency level for different motor locations (front, back, left/right shoulder, and left/right waist) are listed in Table 4.

#### 3.4.1. Correlation between Perceived Intensity and Urgency Level

**Figure 8:**
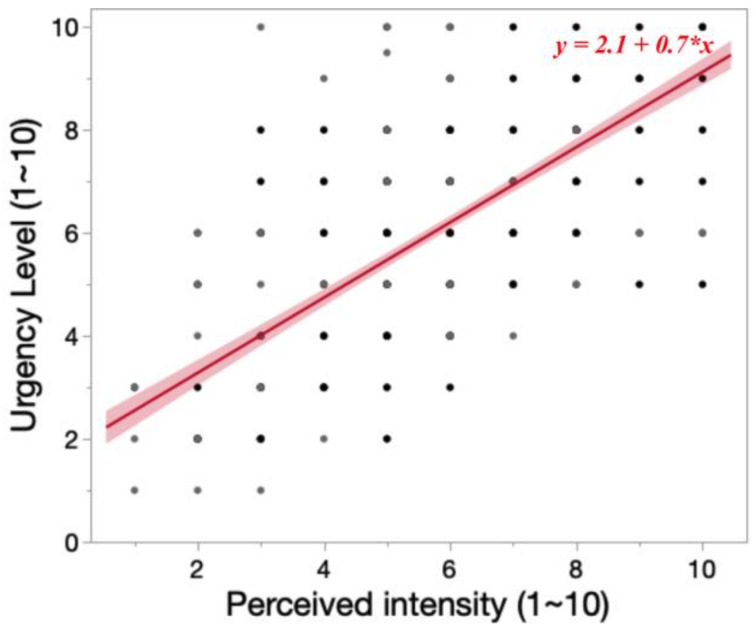
Correlation between perceived intensity and urgency level. Dots in the figure represent collected data points. Darker dots represent multiple data points were overlapped in the figure.

Simple linear regression was used to test if perceived intensity (0~10) significantly predicted urgency level (0~10). As illustrated in Figure 8, the fitted regression model was urgency level = 2.1 + 0.7 × (perceived intensity). The overall regression was statistically significant (R^2^ = 0.53, *F*(1, 478) = 546.54, *p* < 0.0001). It was found that perceived intensity significantly predicted urgency level (*β* = 0.6991, *p* < 0.0001).

#### 3.4.2. Perceived Directional Information

Without any training, 11 out of 17 participants naturally interpreted vibrations as indicating the direction they need to move towards (wayfinders); 4 participants perceived the vibrations as signaling a hazard present in that direction (warnings); and 2 participants had mixed perceptions, treating some vibrations as wayfinders and others as warnings.

### 3.5. NASA-TLX Workload Ratings

**Figure 9:**
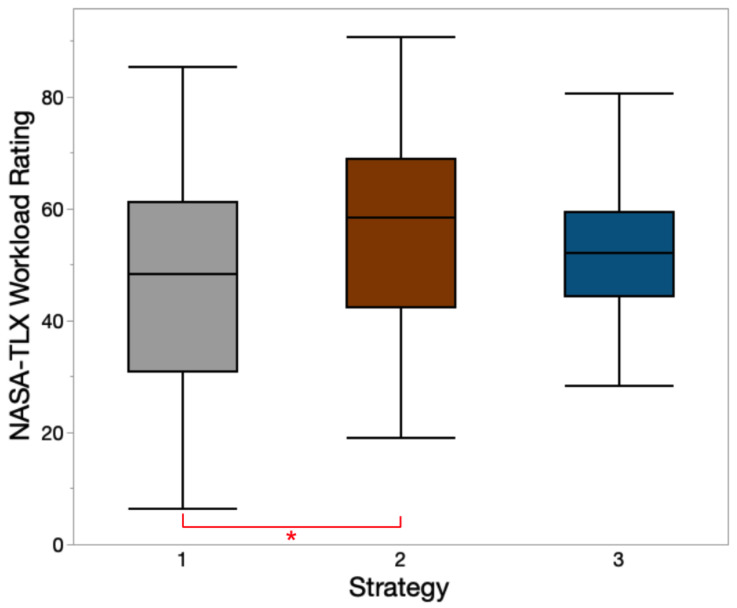
NASA-TLX workload rating for different strategies. * indicates significant pairwise Tukey HSD comparisons (*p* < 0.05) among these notification strategies.

A total of 102 trials (17 participants × 6 trials/participant) were conducted in this task. Due to the instrument errors, only 96 trials (16 participants × 6 trials) were analyzed. The mean ± SD values of NASA-TLX weighted workload ratings for strategies 1, 2, and 3 were 47.9 ± 20.6, 56.6 ± 16.5, and 52.6 ± 13.5, respectively. Notification strategy had a significant main effect on the workload rating (*F*(2, 78) = 9.59, *p* = 0.008). Tukey HSD post hoc tests indicated strategy 1 had a significantly lower workload rating than strategy 2. A box plot of workload ratings for three strategies is presented in Figure 9.

### 3.6. Usability Ratings

A total of 17 survey results were collected from 17 participants. The mean ± SD values of comfort level (0~10), effectiveness (0~10), and willingness to use the strategy (0~10) for strategy 1 were 3.3 ± 2.7, 2.9 ± 2.8, 6.5 ± 2.6, and 6.4 ± 2.5, respectively. The mean ± SD values of comfort level (0~10), effectiveness (0~10), and willingness to use the strategy (0~10) for strategy 2 were 4.7 ± 2.7, 4.6 ± 2.5, 6.2 ± 1.9, and 5.4 ± 2.8, respectively. The mean ± SD values of comfort level (0~10), effectiveness (0~10), and willingness to use the strategy (0~10) for strategy 3 were 4.1 ± 2.8, 4.5 ± 2.6, 6.2 ± 2.6, and 5.9 ± 2.8, respectively. No significant differences were found between notification strategies and all three subjective measurements.

**Figure 10:**
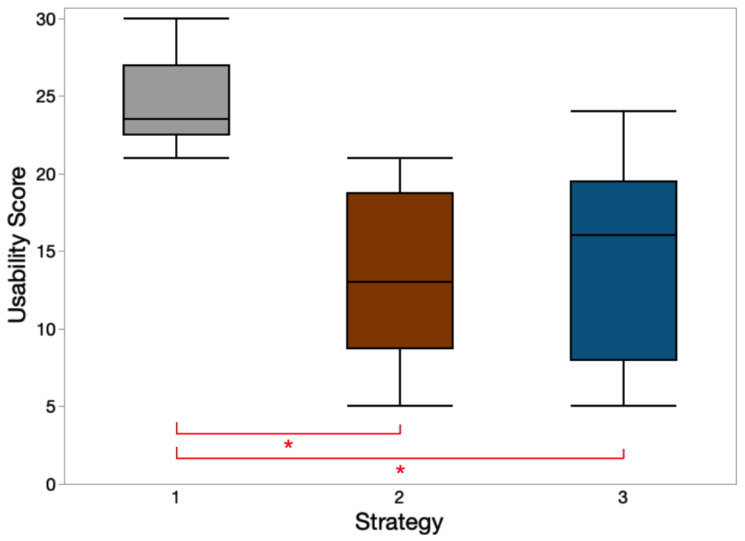
Usability score for different strategies. * indicates significant pairwise Tukey HSD comparisons (*p* < 0.05) among these notification strategies.

The mean ± SD values of usability scores were 24.5 ± 3.1, 13.3 ± 5.7, and 14.7 ± 6.8 for strategies 1, 2, and 3, respectively. A Kruskal–Wallis test indicated that the usability score differed over strategies; χ^2^(2) = 9.18, *p* = 0.010. Post hoc tests indicated that strategy 1 had a significantly higher usability score than strategies 2 and 3. A box plot of usability scores for three strategies is presented in Figure 10.

### 3.7. Preferred Strategy

For the preferred notification strategy, the responses from 17 participants were almost evenly distributed: 6 participants preferred strategy 1 (providing a moving direction), 5 participants preferred strategy 2 (providing a hazard direction), and 6 participants preferred strategy 3 (providing a hazard direction and a moving direction).

### 3.8. Preferred Strategy and Perceived Directional Information

The number of participants for the “preferred strategies” and “perceived directional information” is displayed in Table 5.

## 4. Discussion

### 4.1. Reaction Time

A complete hazard prevention system usually consists of three subsystems: a hazard detection system, a signal transmission system, and a warning system. The proposed vibrotactile alerting system, a subtype of the warning system, serves as the ultimate safeguard separating workers from potential hazards. Gaining insight into the reaction time of the warning system is pivotal for the strategic design and configuration of other parts of other segments of the hazard prevention system. Given that the average reaction time for users to respond to vibrotactile signals was 337 ms, one can calculate the minimum safe distance required between the worker and the hazard, so workers can take timely action and prevent hazards. This calculation can be derived using the following equation:

(Safe distance between workers and hazard at the time of alerting) =  (reaction time) × (speed of approaching hazard).(1)

Table 6 provides examples of two speeds of approaching hazards and their corresponding safe distances. When contrasted with findings from Mark et al. [69], which suggest that the minimum safe distance should exceed 10.61 m and 34 m for vehicle speeds of 40 km/h and 72 km/h, respectively, the proposed system appears to reduce the required minimum safe distance by approximately 64.8% and 80.2%.

In an earlier study from Woodworth and Schlosberg [70], it was found that the reaction time for auditory stimuli was about 30 to 50 ms faster than visual stimuli due to slower sensory processing in vision. However, results from this study revealed that in noisy and complex environments such as highway construction work zones, both vibrotactile and visual signals received significantly faster reaction times than audio signals. The average reductions were 43.6% and 25.8% for vibrotactile and visual signals, respectively. Participants reported that the main issue with audio signals was the hearability, especially when they were focusing on the contents of the 360° video. This finding indicates that the environment and working conditions have a significant impact on users’ reaction times to different alerts, and they should be taken into account when designing hazard prevention interventions for workers working in dynamic work environments.

Despite the lack of significant differences found in reaction times between vibrotactile and visual signals, several participants reported that visual signals were difficult to discern when their attention was not directly focused on them. Similarly, warning lights in construction sites can be masked by ambient light during the day, making perception hard for workers. On the other hand, visual warnings may be better perceived during night shifts, which are very common in highway construction. Meanwhile, as mentioned by Burke et al. [71], combining multiple sensory modalities may improve the performance of receiving signals. Therefore, future studies should be conducted in the construction field to evaluate the reaction time of combinatory sensory modalities.

As noted in research by Johns and Sarter [40], different body locations have varying sensitivities, vibratory thresholds, and spatial acuities. This study demonstrated that the same vibration can be perceived with different intensities at different body locations (Table 1 and Table 2). Since motor location has no significant effect on reaction time, a vibrotactile warning system can place vibration actuators at various locations as long as the vibration is noticeable by users. However, considering the limitations of adding sensors to workers’ PPE as mentioned before, the warnings activated on the upper trunk, i.e., sternum, back, and shoulders, may be better perceived compared to the waist, which is used in other methods.

### 4.2. Correctness

In phase 2, when the motors were dispersed across various body locations, all 17 participants accurately recognized directional cues linked to activated vibration motors with 100% correctness. Unlike the study by Sakhakarmi et al. [44], where all vibrotactile cues were provided at the lower backs of participants, this study was able to compare the results for different body parts, allowing more practical configuration in the future. Their results displayed a correctness range of 98% to 100% in identifying directional cues among five participants. Detailed comparisons are presented in Table 7. These findings suggest that the proposed system demonstrates promising mapping between directional cues and the location of vibration motors.

In phase 3, when the primary work tasks were included, the average correctness of responding to warning messages was decreased to 95.77% from the 100% accuracy achieved in phase 2, in which participants were only tasked to identify directional cues from warning messages.

Several factors may have contributed to this reduction in correctness. Firstly, introducing the primary task significantly increased the workload as participants were required to mimic a worker’s movements. However, the observed decrease in correctness underscores the necessity of considering realistic working conditions to effectively assess the performance of alerting systems. Such conditions could encompass the consideration of interruption and task-switching processes [24].

### 4.3. Perception of Vibrations

As expected, the mean perceived intensity and urgency level increased by 47.9% and 43.1%, respectively, as the motor intensity increased from 50% to 100%. In a previous study, White [64] obtained a similar result when increasing the motor intensity from 12 dB to 23.5 dB: the mean perceived urgency rating (0~10) increased from 3.24 to 6.25. Regarding pulse rate, the results from this study showed that increasing the pulse length and inter-pulse interval increased both the perceived intensity and urgency level. In contrast, White [64] and Pratt et al. [63] discovered that increasing the inter-pulse interval decreased the perceived urgency. This discrepancy suggests further research is needed to understand how pulse length and inter-pulse interval influence the perceived intensity and urgency level.

Perceived intensities and urgency levels on the left and right waists were significantly lower than those on the shoulders, likely due to the loose contact between vibration motors and waists—an issue inherent in the current hardware design. One potential solution is to attach a suspension frame to vibration motors and secure the frame tightly to the waists [72]; however, this could significantly increase the system’s weight and affect the comfort level. Therefore, providing vibrotactile signals on the left and right shoulders might be a better alternative.

The strong correlation between perceived intensity and urgency level indicates that by altering the intensity of vibrations, a vibrotactile system can convey urgency information to users. For instance, when a hazard approaches, the system can gradually increase the intensity to notify the user by mapping the urgency level to the distance between the user and the hazard. However, given that the rated vibration intensity varies from motor type, a motor with a higher maximum vibration intensity might deliver more levels of information. Further research is needed to understand the minimal distinguishable intensity change on the skin for different motor types.

The results regarding perceived vibration information indicate people may have different natural responses to vibrations. Although the majority of participants (11 out of 17) in this study naturally felt vibrations indicating a direction they need to move towards (wayfinder), it is premature to conclude that mapping a movement direction with vibrations is the optimal notification strategy. A customized notification strategy, based on each user’s intuitive response to vibrotactile signals, might help increase the adaptability of the system to different users. When customization is restricted by hardware constraints, training could potentially help alter a user’s response to vibrations. However, further research is needed to understand the training effect on the user’s response to vibrotactile signals.

### 4.4. Preferred Notification Strategy

Although notification strategy 1 had a significantly lower workload rating and higher usability score than strategy 2, this does not necessarily imply that strategy 2 is substantially inferior to strategy 1. The evenly distributed preferences among participants (six favored strategy 1, five favored strategy 2, and six favored strategy 3) indicate that multiple factors may influence the strategy preference. Based on the post-experiment interviews, participants who preferred strategy 1 mentioned that it was “the most intuitive” strategy, requiring minimum effort to respond to the vibrotactile signals. Those who preferred strategy 2 felt the vibrations were like warnings, signaling that they need to “get away from” the hazard. Participants who preferred strategy 3 appreciated it because it provided more information, which naturally aligned with their reaction to a hazard: first, locate the hazard, and then move away from it. This finding suggests people may have different mental models of processing vibrotactile signals [73], leading to contrasting reactions. Considering that the majority of participants (11 out of 17) naturally treated vibrations as a wayfinder, future between-group studies could investigate the factors affecting the preference for different strategies.

## 5. Conclusions

This study proposed a vibrotactile system embedded in a safety vest, designed to provide alerts and guidance towards safe areas when a hazard approaches. By considering multiple human factors, this study evaluated reaction time, the perception of vibrotactile signals, the workload of different notification strategies, and the usability of the proposed system.

The first phase of this study investigated the feasibility of using vibrotactile signals as an alerting cue in highway construction work zone environments. Comparing reaction times between different sensory modalities revealed that the mean reaction times of vibrotactile and visual signals were decreased by 43.6% and 25.8% from audio signals (597 ms), respectively. This result suggests that vibrotactile signals may be more feasible for providing warnings in noisy and complex environments.

The second phase of this study explored the perception and performance of vibrotactile signals activated at different body locations. When increasing the vibration motor intensities from 50% to 100% or pulse/inter-pulse lengths from 60 ms to 400 ms, participants’ perceived intensities and urgency levels significantly increased. This study also found the left and right waists had significantly lower perceived intensities and urgency levels than all other locations, suggesting that the left and right shoulders may be better locations for placing vibration motors. A high correlation was observed between perceived intensity and urgency level, indicating that the urgency of a hazard can be mapped with vibration intensity.

The third phase of this study investigated the usability of three different notification strategies in drawing and walking tasks. Participants’ workload, comfort level, effectiveness, and willingness to use the strategy showed strategy 1 had the lowest workload and highest usability score. However, among 17 participants, 6 preferred strategy 1, 5 preferred strategy 2, and 6 preferred strategy 3. This finding indicates that there might not be an optimal notification strategy that would suit all users, and system customizability may be necessary to ensure high usability.

In this preliminary study, various human factors were considered when designing and evaluating the proposed vibrotactile system. However, the limited number of tasks may not represent all types of work in highway work zones, and also, the long-term effect of vibrotactile systems on users remains to be revealed. Further studies with various tasks should be conducted to evaluate whether the system can be practically applied to diverse working conditions with longer working periods. Simultaneously, given that this research was conducted in a laboratory setting, the impact of adverse weather conditions, such as temperature fluctuations, wind, and humidity, on the system performance remains unexplored. Future field-based studies will be conducted to assess the system’s resilience and performance against environmental interferences. Such a system, with consideration of human factors, could play an essential role in improving workers’ hazard awareness in highway construction work zones.

## Figures and Tables

**Table 1:** Reaction time of different motor locations (60 ms pulse length).

	Motor Location					
	Front	Back	Left		Right	
			Shoulder	Waist	Shoulder	Waist
Reaction Time (ms)	528 ± 326	443 ± 169	480 ± 187	462 ± 221	494 ± 227	545 ± 479

**Table 2:** Reaction time of different motor locations (400 ms pulse length).

	Motor Location					
	Front	Back	Left		Right	
			Shoulder	Waist	Shoulder	Waist
Reaction Time (ms)	621 ± 357	565 ± 330	562 ± 243	508 ± 206	515 ± 246	553 ± 390

**Table 3:** Subjective ratings of different vibration settings.

	Motor Intensity		Pulse/Inter-Pulse Length	
	50%	100%	60 ms	400 ms
Perceived Intensity (0~10)	4.8 ± 2.0 ^a^	7.1 ± 2.1 ^b^	5.1 ± 2.2 ^a^	6.8 ± 2.1 ^b^
Urgency Level (0~10)	5.1 ± 2.0 ^a^	7.3 ± 1.9 ^b^	5.9 ± 2.4 ^a^	6.4 ± 2.2 ^b^

^a, b^ The superscripts indicate significant differences between results.

**Table 4:** Subjective ratings of different motor locations.

	Motor Location					
	Front	Back	Left		Right	
			Shoulder	Waist	Shoulder	Waist
Perceived Intensity (0~10)	6.3 ± 2.3 ^a^	6.4 ± 2.2 ^a^	6.3 ± 2.4 ^a^	5.2 ± 2.3 ^b^	6.1 ± 2.5 ^a^	4.7 ± 2.3 ^b^
Urgency Level (0~10)	6.5 ± 2.1 ^a^	6.7 ± 2.0 ^a^	6.5 ± 2.3 ^a^	5.7 ± 2.4 ^b^	6.1 ± 2.3 ^a^	4.9 ± 2.4 ^b^

^a, b^ The superscripts indicate significant differences between results.

**Table 5:** Distribution of perceived directional information across preferred strategies in the study population.

Preferred Strategy (Number of Participants)	Perceived Vibration Information		
	Wayfinder	Warning	Mixed
Strategy 1 (6)	5	1	0
Strategy 2 (5)	1	2	2
Strategy 3 (6)	5	1	0

**Table 6:** Speed of approaching hazard and the corresponding minimum safe distance between the worker and the hazard.

Hazard Approaching Speed	Minimum Safe Distance
40 km/h	3.74 m
72 km/h	6.74 m

**Table 7:** Results comparison with Sakhakarmi et al. [44].

System	Smart Vest	System from Sakhakarmi et al. [44]
Number of participants	17	5
Number of total trials	1088	1250
Average correctness	100%	99.12%

## Data Availability

The data presented in this study are available on request from the corresponding author. The data are not publicly available due to the privacy policy of Virginia Tech.
